# Effects of Bariatric Surgery on Human Small Artery Function: Evidence for Reduction in Perivascular Adipocyte Inflammation, and the Restoration of Normal Anticontractile Activity Despite Persistent Obesity

**DOI:** 10.1016/j.jacc.2013.04.027

**Published:** 2013-07-09

**Authors:** Reza Aghamohammadzadeh, Adam S. Greenstein, Rahul Yadav, Maria Jeziorska, Salam Hama, Fardad Soltani, Phil W. Pemberton, Basil Ammori, Rayaz A. Malik, Handrean Soran, Anthony M. Heagerty

**Affiliations:** ∗ Cardiovascular Research Group, University of Manchester, Manchester, United Kingdom; † Manchester Wellcome Trust Clinical Research Facility, Manchester, United Kingdom; ‡ Department of Clinical Biochemistry, Manchester Royal Infirmary, Manchester, United Kingdom; § Salford Royal NHS Foundation Trust, Manchester, United Kingdom

**Keywords:** bariatric, cardiovascular, obesity, AdipoR1, adiponectin receptor 1, BMI, body mass index, hsCRP, high-sensitivity C-reactive protein, NO, nitric oxide, PVAT, perivascular adipose tissue, TNF, tumor necrosis factor

## Abstract

**Objectives:**

The aim of this study was to investigate the effects of bariatric surgery on small artery function and the mechanisms underlying this.

**Background:**

In lean healthy humans, perivascular adipose tissue (PVAT) exerts an anticontractile effect on adjacent small arteries, but this is lost in obesity-associated conditions such as the metabolic syndrome and type II diabetes where there is evidence of adipocyte inflammation and increased oxidative stress.

**Methods:**

Segments of small subcutaneous artery and perivascular fat were harvested from severely obese individuals before (n = 20) and 6 months after bariatric surgery (n = 15). Small artery contractile function was examined in vitro with wire myography, and perivascular adipose tissue (PVAT) morphology was assessed with immunohistochemistry.

**Results:**

The anticontractile activity of PVAT was lost in obese patients before surgery when compared with healthy volunteers and was restored 6 months after bariatric surgery. In vitro protocols with superoxide dismutase and catalase rescued PVAT anticontractile function in tissue from obese individuals before surgery. The improvement in anticontractile function after surgery was accompanied by improvements in insulin sensitivity, serum glycemic indexes, inflammatory cytokines, adipokine profile, and systolic blood pressure together with increased PVAT adiponectin and nitric oxide bioavailability and reduced macrophage infiltration and inflammation. These changes were observed despite the patients remaining severely obese.

**Conclusions:**

Bariatric surgery and its attendant improvements in weight, blood pressure, inflammation, and metabolism collectively reverse the obesity-induced alteration to PVAT anticontractile function. This reversal is attributable to reductions in local adipose inflammation and oxidative stress with improved adiponectin and nitric oxide bioavailability.

Obesity has become a global public heath challenge affecting almost one-half billion adults (1) and an estimated 40 million children (2). Large artery disease is very common in obese patients and manifests clinically as myocardial infarction, stroke, and hypertension (3). Small artery disease is also common in obesity and contributes to the development of hypertension and microvascular disease due to changes in peripheral resistance and local autoregulation (4). Historically, the small artery dysfunction in obesity has been attributed to damage to the endothelium (5), most notably to generation and release of nitric oxide (NO) (6). More recently, however, appreciation has grown for an additional mechanism by which vascular damage occurs in obesity; the function of fat surrounding arteries, or perivascular adipose tissue (PVAT). The PVAT surrounds the majority of blood vessels in the body and, in addition to adipocytes, contains inflammatory cells, stem cells, and microvasculature. In health, PVAT modulates the contractile tone of adjacent small arteries by secreting vasodilatory molecules. These adipose-derived vasodilators act independently of the endothelium and include adiponectin (7), NO (7), hydrogen sulfide (8), and palmitic acid methyl ester (9). In 2009, we performed the first human small artery study of PVAT and showed that subcutaneous gluteal PVAT from lean healthy individuals reduced adrenergic constriction in adjacent arteries, an “anticontractile” effect. However, in patients with metabolic syndrome the vasodilatory effect of the PVAT was entirely lost, due to dual processes of adipose tissue hypoxia and inflammation, both of which are established sequelae of obesity in fat depots (7). More recently, we have seen that macrophage activation in adipose tissue contributes to the attenuation in PVAT anticontractile effect (10).

Bariatric surgery has been performed for nearly 60 years and is established as the most effective clinical intervention to achieve both significant and sustained weight-loss in severely obese individuals. There are 3 types of bariatric surgical procedures: restrictive, malabsorptive, and combined operations (11). Gastric bypass surgery is a combination of restriction and malabsorption and has been shown to achieve a significantly higher degree of weight loss than restrictive bariatric surgery (12).

Bariatric surgery also dramatically improves cardiovascular risk profiles in obese patients and reduces overall mortality 13, 14. The mechanisms that underlie these cardiovascular improvements remain unclear, however. In the present study we investigated the effects of bariatric surgery achieved by the gastric bypass method on the vasodilatory properties of PVAT. We report amelioration of inflammation in PVAT with complete restoration of anticontractile activity as a consequence of improved adiponectin and NO bioavailability, despite persisting obesity.

## Methods

### Study population

Patients with severe obesity (body mass index [BMI] >35 kg/m^2^) who were awaiting gastric bypass surgery (n = 15) and lean healthy volunteers (n = 7) were recruited after full informed written consent in accord with local research ethics committee approval. All participants provided a fasting venous blood sample for the measurement of inflammatory markers and adipokines.

The study participants also provided gluteal subcutaneous fat samples (1.5 × 1.5 × 1.5 cm) by undergoing a surgical biopsy under local anesthesia (7). The sample was immediately processed in 3 sections. One part was stored for immunohistology, the second was snap-frozen for estimation of NO levels, and the remainder was used to harvest small subcutaneous arteries with micro-dissection.

Blood pressure was recorded as a mean of 3 recordings measured with a semi-automated machine (OMRON 705CP, White Medical, Clifton-Upon-Dunsmore, United Kingdom), whereas the participants were seated at rest for 15 min.

Further measurements including BMI and waist circumference were also recorded.

The obese patients were invited to return for a follow-up assessment, including a biopsy, 6 months after bariatric surgery.

### Biochemical analyses

High-sensitivity C-reactive protein (hsCRP) was measured in serum by an in-house, antibody sandwich enzyme-linked immunoadsorbent assay technique with anti-human CRP antibodies, calibrators, and controls from Abcam (Cambridge, United Kingdom). Interleukin-6, tumor necrosis factor (TNF)-α, adiponectin, leptin, and resistin were measured in serum, and E-selectin was measured in plasma, all with DuoSet ELISA development kits from R&D Systems (Abingdon, United Kingdom).

### Wire myography

One section of the gluteal fat biopsy was placed in chilled physiological saline solution (composition in mmol/l: sodium chloride 118, potassium chloride 3.4, magnesium sulphate 1.2, calcium chloride 1, sodium bicarbonate 25, glucose 11, potassium orthophosphate 1.2) and oxygenated. The dissection dish was placed on ice during microdissection to preserve the integrity of the tissue, whereas arterial segments 250 to 350 μm in diameter were harvested, 1 segment with PVAT intact, and the adjacent segment devoid of PVAT. Both segments came from the same artery.

Endothelium denuded vessels were mounted on 40-μm wires and studied with wire myography (Danish MyoTech, Aarhus, Denmark).

The mounted vessel segments were oxygenated and maintained at a temperature of 37°C before vessel diameter and wall tension was normalized as previously described 7, 15. Vessels were challenged with a 60-mmol/l high-potassium physiological saline solution to establish viability and baseline constriction.

Each vessel segment was stimulated with cumulative doses of norepinephrine (Sigma-Aldrich, Dorset, United Kingdom) at the following doses: 10^−9^; 10^−8^; 3 × 10^−8^; 10^−7^; 3 × 10^−7^; 5 × 10^−7^; 10^−6^; 2 × 10^−6^; 3 × 10^−6^; 5 × 10^−6^; 10^−5^; 2 × 10^−5^; and 3 × 10^−5^ mol/l. Contractile responses to norepinephrine are presented as a percentage of high-potassium physiological saline solution constriction, consistent with published data 7, 16, 17, 18.

### Pharmacological assessment

Pharmacological protocols were applied to study the effect of PVAT on adjacent small arteries. In each case 2 segments of the same artery were prepared with and without PVAT attached as previously described (7).

The role of oxidative stress was evaluated by incubation of samples from pre-operative patients with superoxide dismutase and catalase (superoxide dismutase, Sigma-Aldrich, 100-μ/ml incubation period 45 min; and catalase, Sigma-Aldrich, 100-μ/ml incubation period 45 min; n = 4).

Further protocols assessed the contribution of NO and adiponectin to PVAT function in samples taken from patients after weight loss. Arteries with PVAT were incubated with blocking peptide for adiponectin receptor 1 (AdipoR1) (5 μg/ml, 1.6 × 10^−4^ mol/l, Enzo Life Sciences; 45 min; n = 7) and N5-[imino(methylamino)methyl]-L-ornithine, citrate (5 × 10^−5^ mol/l, Sigma; 45 min; n = 4).

### NO assay

Five paired pre-operative and post-operative frozen PVAT samples were homogenized by suspending the tissue in 400 μl of lysis buffer (50 mmol/l Tris base, 150 mmol/l sodium chloride, 2 mmol/l ethylenediaminetetraacetic acid, 2 mmol/l ethyleneglycoltetraacetic acid, 40 mmol/l β-glycerophosphate, 50 mmol/l sodium fluoride, 10 mmol/l sodium pyrophosphate, 10% glycerol, 1% Triton X-100 [pH 7.4]) and protease inhibitor (Complete Mini EDTA-Free; Roche Diagnostics, Basel, Switzerland) on ice and using a Dounce (glass/glass) tissue grinder set (Sigma-Aldrich). Upon complete homogenization, the solution was incubated at 4°C for 10 min, then centrifuged for 10 min at 16,000 *g*. The resulting supernatant was then removed for application of the assay.

Total nitrate/nitrite concentration was measured with a NO colorimetric assay kit according to the instructions of the manufacturer (Abcam).

### Immunohistochemistry

#### Macrophage Markers and TNF-α

Immediately after dissection, tissue from each gluteal biopsy consisting of skin and subcutaneous fat was placed in 4% paraformaldehyde in phosphate buffered saline for 24 h and subsequently processed to paraffin wax blocks. Consecutive 5-μm sections were de-waxed, rehydrated, and immunostained for 2 macrophage markers—CD68 [KP1] (Dako, Glostrup, Denmark; dilution 1:100), and CD68 [PGM1] (Biocare Medical, Concord, California; dilution 1:100)—and the pro-inflammatory cytokine TNF-α (Abcam; dilution 1: 100). Antigen retrieval was performed, followed by blocking of endogenous peroxidase and nonspecific protein binding with Dako blocking solutions. Tissue sections were incubated with primary antibodies for 18 h, followed by anti-rabbit/anti-mouse EnVision-HRP (Dako) and finally by Vector SG chromogen kit (Vector Laboratories, Burlingame, California), used to disclose the presence of the macrophages and TNF-α. Color images were captured with a Go-3 QImaging camera (QImaging Corporation, Vancouver, Canada) mounted on a Leitz Diaplan microscope (Leica, Wetzlar, Germany). Cells stained with macrophage markers present in adipose tissue were counted, and the results were expressed as cells/mm^2^. For assessment of TNF-α, quantitative analysis of immunostaining was obtained, converting color images to greyscale and with a macro subroutine in an ImagePro version 6.2 image analysis program (MediaCybernetics United Kingdom, Marlow, United Kingdom). The extent of staining was expressed as a percentage of the entire area photographed. Adipocyte size was quantified on microphotographs obtained on Zeiss Axio Imager M2 microscope equipped with AxioCam camera and AxioVision Rel. 4.8 program by free-hand tracing the margins of 100 consecutive cells (on average)/case to avoid selection bias (total: 3,000 cells).

### Immunohistochemistry for AdipoR1

Five μm deparaffinized formalin-fixed tissue sections were microwaved in citrate buffer pH 6.0, treated with 0.3% hydrogen peroxide, blocked with Protein Block (Dako), and incubated with 1 of the anti-AdipoR1 antibodies, goat polyclonal to AdipoR1 (Abcam), or a rabbit monoclonal to AdipoR1 (Epitomics, Burlingame, California), followed by anti-rabbit/anti-mouse EnVision-HRP (Dako) and Vector SG chromogen kit (Vector Laboratories). The results of immunostaining were compared, and the monoclonal rabbit antibody was selected for further use. Slides were examined and images captured on a Zeiss Axio Imager M2 microscope.

### Statistical analysis

The statistical presentation includes paired and unpaired tests. In the case of ordinal tests, medians and quartiles are used, and for parametric tests mean ± SD is used. Cumulative concentration-response curves were constructed with data obtained by wire myography and analyzed with a 2-way analysis of variance and a Bonferroni post hoc test for each dose. A p value of <0.05 was considered statistically significant. Analyses were performed with the GraphPad Prism 5 software (GraphPad, La Jolla, California).

## Results

### Study design and participants

Six months after surgery and weight loss, the patients had a significantly lower waist circumference (n = 15; p < 0.0001), BMI (p < 0.0001), and systolic blood pressure (p = 0.0025) (Table 1).

Post-operatively, there was a significant reduction in insulin (p = 0.0042), fasting glucose (p = 0.0312), and hemoglobin A1c (p < 0.0071) and an improvement in pancreatic beta-cell function (homeostatic model assessment of beta-cell function; p < 0.01) and insulin resistance (homeostasis model assessment of insulin resistance; p = 0.0023). After surgery serum levels of adiponectin increased significantly (p = 0.0197), and leptin decreased (p = 0.0014), but resistin levels did not change significantly (p = 0.0796).

### Weight-loss surgery restores PVAT anticontractile function

**Figure 1:**
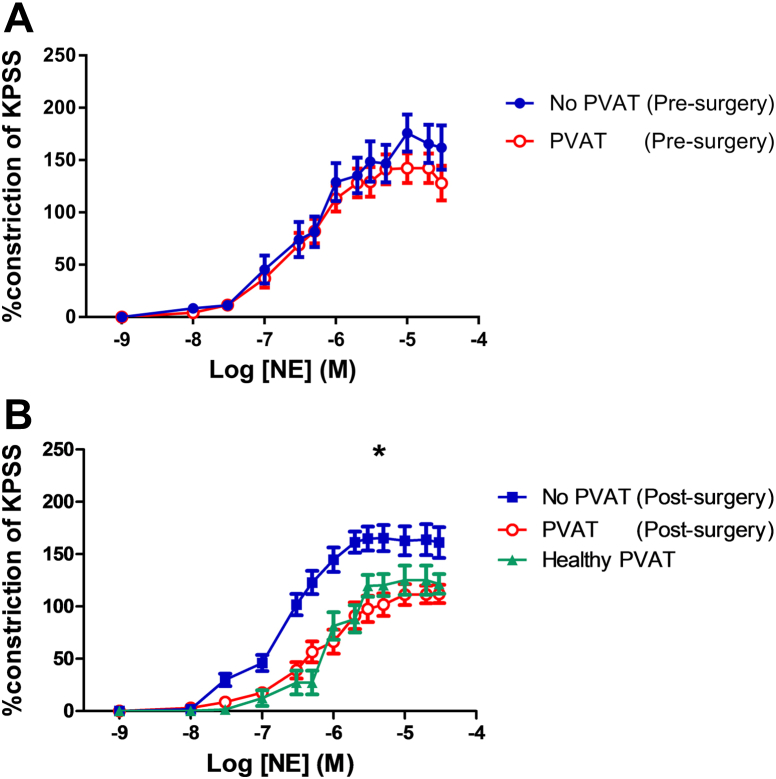
Effect of PVAT on Small Artery Tone Before and After Surgery **(A)** In pre-surgery patients, the presence of perivascular adipose tissue (PVAT) did not affect vessel contractility as compared with skeletonized segments of the same vessel (n = 20, p = 0.95). **(B)** In post-surgery patients, the presence of PVAT had a significant anticontractile effect on the small artery contractility (n = 15, ∗p < 0.01). KPSS = high-potassium physiological saline; Log [NE] (M) = log of the molar concentration of norepinephrine.

In severely obese patients before surgery and weight-loss, PVAT did not significantly alter the norepinephrine-induced contractility of the small arteries in comparison with skeletonized segments of the same vessels (n = 15, p = 0.96) (Fig. 1A).

In samples taken from patients 6 months after surgery and weight loss, PVAT had a significant anticontractile effect on vessels when compared with vessels without PVAT (n = 15, p < 0.01) (Fig. 1B). The vessels with intact PVAT after surgery had a very similar response to cumulative doses of norepinephrine as the PVAT-intact vessels harvested from healthy lean volunteers (p = 0.52, n = 7) (Fig. 1B). The maximal degree of constriction is lower in PVAT-intact vessels in healthy and post-surgery samples as compared with skeletonized vessels, but the degree of constriction of skeletonized vessels to norepinephrine is similar in segments from pre- and post-surgery (Online Fig. 1).

### Anticontractile property of PVAT after weight loss is abolished by adiponectin blockade

**Figure 2:**
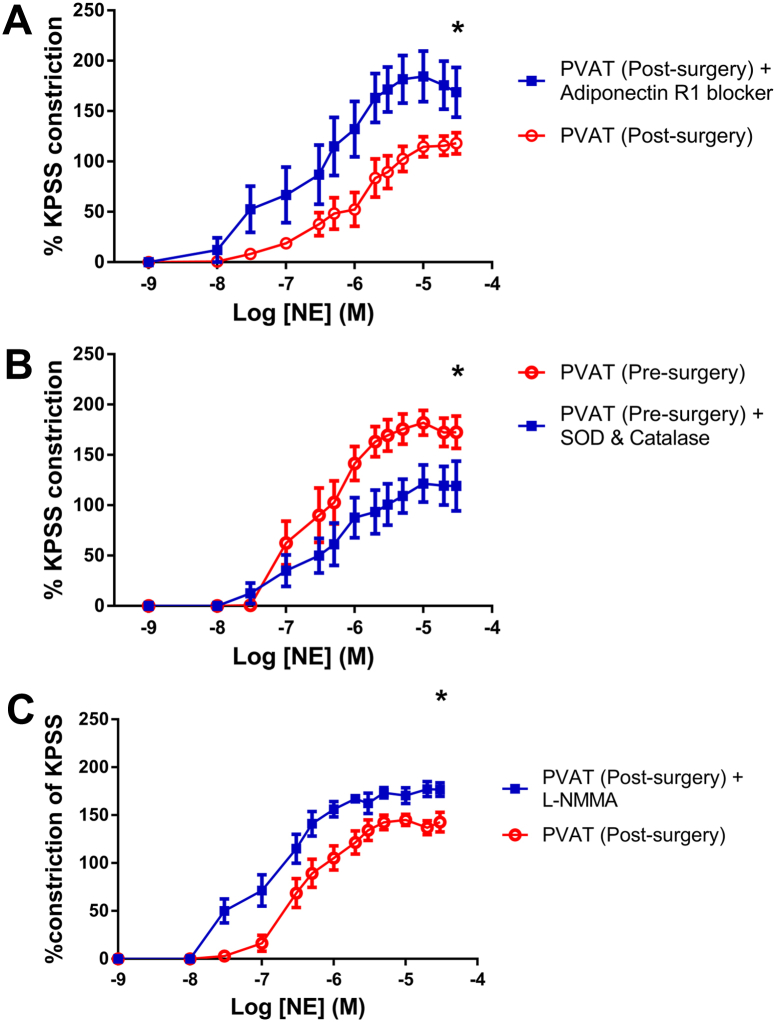
Pharmacological Protocols on PVAT Pre-Surgery and Post-Surgery **(A)** Blocking peptide for polyclonal antibody to adiponectin receptor 1 increases vessel contractility to norepinephrine (n = 7, p <0.0001). **(B)** Incubation with superoxide dismutase (SOD) and catalase rescues the PVAT anticontractile effect in samples taken from pre-surgery patients (n = 7, p < 0.001). **(C)** Post-surgery, inhibition of nitric oxide synthase by incubation with N5-[imino(methylamino)methyl]-L-ornithine, citrate (L-NMMA) leads to increased contractility of vessel segments with intact PVAT (n = 4, ∗p < 0.001). Abbreviations as in Figure 1.

Samples taken from patients 6 months after surgery were incubated with blocking peptide for AdipoR1. This had no effect on segments without PVAT, but in vessels with PVAT there was a significant increase in vessel contractility (p < 0.0001, n = 7) (Fig. 2A).

### Free radical scavengers can rescue PVAT anticontractile function in obesity

In patients with severe obesity, the presence of perivascular adipose tissue had no effect on vessel contractility in comparison with vessel segments devoid of PVAT. Incubation of the vessels with the free radical scavengers superoxide dismutase and catalase resulted in a shift in the curve to resemble that of vessels with intact PVAT taken from healthy individuals (p < 0.001, n = 7) (Fig. 2B).

### Increased NO bioavailability after surgery

There was a significant increase in NO in PVAT of severely obese patients 6 months after surgery (1.016 vs. 1.196 nmol/μl, p = 0.029, n = 5).

In post-surgery samples, de-endothelialized vessel segments were incubated with N5-[imino(methylamino)methyl]-L-ornithine, citrate. The incubation had no effect on vessels without PVAT, but in vessels with PVAT, there was a significant increase in contractility to cumulative doses of norepinephrine (p < 0.001, n = 4) (Fig. 2C).

### Reduced PVAT inflammation, adipocyte size, and AdipoR1 after bariatric surgery

**Figure 3:**
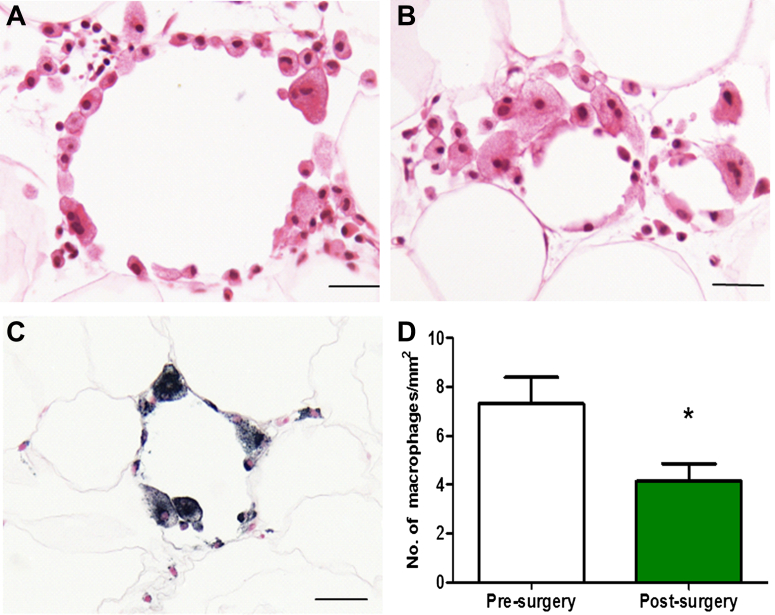
Subcutaneous Adipose Tissue Before and After Surgery Inflammatory infiltrates composed mostly of macrophages were present in wreath-like arrangements **(A)**, in groups **(B)**, or were scattered. Staining for macrophage marker (CD68 (KP1) **(C)** allowed for performing cell counts. There was a significant reduction in the number of CD-68 staining macrophages post-surgery (n = 14, 7.3 ± 1.1 vs. 4.1 ± 0.7, ∗p < 0.01) **(D)**. Scale bar = 20 μm.

**Figure 4:**
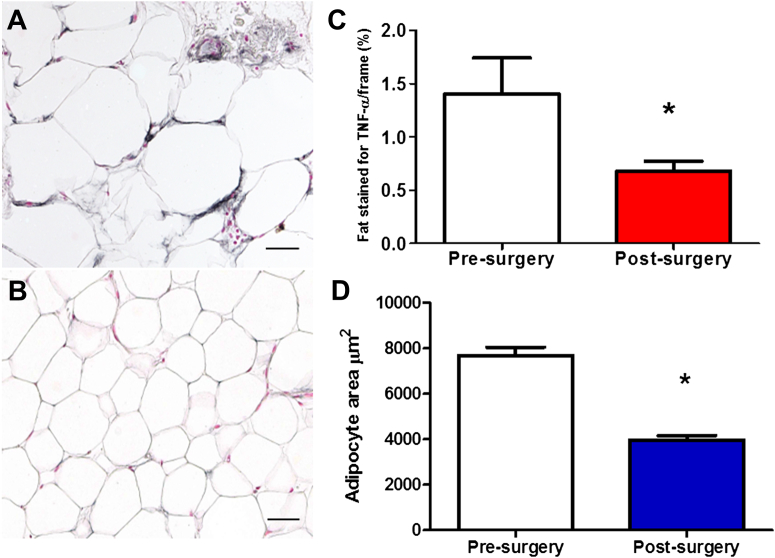
Assessment of TNF-α Staining and Adipocyte Size Pre- and Post-Surgery Immunostaining for tumor necrosis factor (TNF)-α showed that adipocytes themselves were positive for this cytokine, to a higher extent pre-surgery **(A)** than post-surgery **(B)**. Note positive staining of microvessels in the pre-operative biopsy. There is a significant reduction in the percentage of adipose tissue area that stains for TNF-α pre-surgery versus post-surgery (1.41 ± 0.34 vs. 0.68 ± 0.09, ∗p < 0.05) **(C)**. The average adipocyte area was 7,672 ± 369.6 μm^2^ pre-surgery and 3,955 ± 207.5 μm^2^ (∗p < 0.0001) post-surgery **(D)**. Scale bar = 20 μm.

The perivascular adipose tissue was stained, to quantify the inflammation, for both CD68 staining macrophages (Fig. 3) and the cytokine TNF-α (Figs. 4A to 4C).

There was a significant reduction in the CD68 [KP1] staining macrophage numbers after surgery (7.3 ± 1.1 vs. 4.1 ± 0.7, p = 0.0067, n =14) (Figs. 4C and 4D), but the change in CD68 [PGM1] staining was not statistically changed. There was a significant reduction in the percentage of adipose tissue area staining for TNF-α (1.41 ± 0.34 vs. 0.68 ± 0.09, p < 0.05) (Figs. 4A to 4C) after surgery.

The average adipocyte area was 7,672 ± 369.6 μm^2^ pre-surgery and 3,955 ± 207.5 μm^2^ (p < 0.0001) post-surgery (Fig. 4D). The change in average adipocyte area correlated with the change in BMI after surgery (R^2^ = 0.356, p = 0.0243, n = 14) (Online Fig. 2).

There was a significant reduction in circulating markers of inflammation including hsCRP (p < 0.001), interleukin-6 (p = 0.013), monocyte chemoattractant protein-1 (283.1 vs. 258.3 pg/ml; p = 0.013), and the adhesion molecule E-selectin (12.7 vs. 7.47 ng/ml; p < 0.001). However, there was no significant change in circulating TNF-α levels (p = 0.519) (Table 1).

**Table 1:** Patient Demographic Data and Biomarker Profile

	Healthy	Pre-Surgery	Post-Surgery	p Value
Age, yrs	51.0 ± 11.5	53.2 ± 6.73	53.2 ± 6.73	
Waist, cm	92.0 ± 11.6	140.90 ± 14.85	115.5 ± 13.3	<0.0001
BMI, kg/m^2^	25.40 ± 2.68	51.58 ± 7.47	37.960 ± 7.175	<0.0001
Systolic BP, mm Hg	135.0 ± 17.2	146.1 ± 28.8	132.70 ± 22.15	0.0025
hsCRP, mg/l	1.33 ± 1.09	6.321 ± 2.444	2.747 ± 2.282	0.0027
IL-6, pg/ml	2.070 ± 0.811	2.830 ± 1.755	0.644 ± 0.774	0.0052
TNF-α, pg/ml	4.83 ± 1.75	35.14 ± 40.30	27.14 ± 37.30	0.12
Adiponectin, mg/l	2.190 ± 0.633	1.845 ± 0.699	2.410 ± 0.828	0.011
Leptin, ng/ml	9.67 ± 7.76	59.9 ± 17.7	24.91 ± 10.42	0.0014
Resistin, ng/ml	9.26 ± 3.05	15.780 ± 6.823	13.550 ± 4.605	0.0796
E-selectin, ng/ml	131.0 ± 5.06	12.530 ± 5.581	8.753 ± 3.374	0.0033
Insulin, mU/l	7.18 ± 4.11	27.83 ± 16.17	14.83 ± 10.12	0.004
Glucose, mmol/l	5.080 ± 0.377	6.825 ± 3.585	5.858 ± 2.355	0.0312
HbA1C, %	5.740 ± 0.689	7.0 ± 2.119	6.330 ± 1.603	0.0071
HOMA-B, %	90.2 ± 26.9	243.0 ± 175.7	169.9 ± 143.7	0.01
HOMA-IR, %	0.933 ± 0.557	7.838 ± 4.213	3.860 ± 2.683	0.0023

Values are mean ± SD. The p values compare the matched baseline and post-surgery values and were determined with paired *t* tests.

BP = blood pressure; BMI = body mass index; HbA1C = hemoglobin A1c; HOMA-B = homeostatic model assessment of beta-cell function; HOMA-IR = homeostasis model assessment of insulin resistance; hsCRP = high-sensitivity C-reactive protein; IL = interleukin; TNF = tumor necrosis factor.

## Discussion

**Figure 5:**
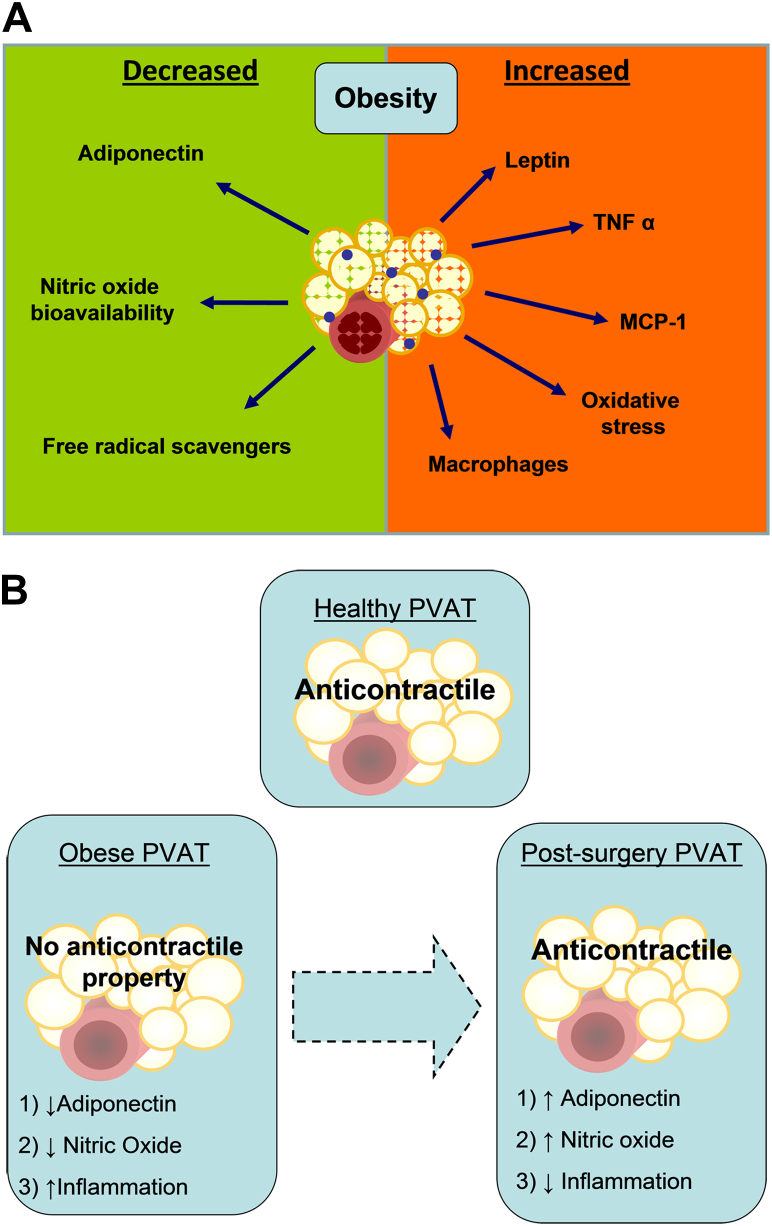
Background and Conclusions **(A)** In obesity there is an increase in perivascular adipose tissue (PVAT) levels of inflammatory cells and cytokines as well as increase in levels of leptin. **(B)** We conclude that, after bariatric surgery, there is an increase in adiponectin levels and nitric oxide bioavailability in PVAT and reduction in tissue inflammation, which contributes to restoration of PVAT anticontractile function. MCP = monocyte chemoattractant protein; TNF = tumor necrosis factor.

The present study investigated the effect of bariatric surgery on the vasodilatory properties of PVAT. We designed the study after our earlier observation that, in severely obese patients, although there is an accumulation of adipose tissue, there is also a paradoxical inhibition of the beneficial PVAT vasodilation (7). There are now 3 main findings presented here. First, bariatric surgery reverses the obesity-induced damage to PVAT anticontractile function. Second, the functional recovery of the PVAT is independent of the endothelium. Third, bariatric surgery restores the function of PVAT by reducing adipose inflammation and increasing local adiponectin and NO bioavailability (Figure 5). The observations advance our understanding of the mechanisms by which obesity disrupts the vasodilating function of adipose tissue and how this pathology might be reversed by clinical intervention.

The cardiovascular complications of obesity are undoubtedly among the most pressing issues facing clinicians today (19). To date, the most common recommended intervention has been the encouragement of dietary modification. Effective and sustained dieting does lead, without doubt, to significant weight loss, but unfortunately the weight loss is not sustained (20). Furthermore, the impact of dietary modification on cardiovascular outcomes is unclear 13, 21. For patients with severe obesity, the most effective method of achieving significant and sustained weight reduction is by weight-reducing surgery (14). In addition, the weight loss after surgery also significantly correlates with improvements to blood pressure 22, 23, left ventricular mass (24), exercise capacity (25), and glucose metabolism (26). Changes to weight in patients are associated with profound modulation of the cytokine and inflammatory profiles of adipose tissue. In this regard, it is now established that, in obese patients, adipose tissue undergoes dual processes of hypoxia and inflammation, leading to a reduction in the secretion of adipocytokines such as adiponectin. The hypoxia is thought to be due to adipocyte hypertrophy (27) twinned with reductions to capillary density and angiogenic capacity (28). This results in up-regulation of hypoxia inducible factor-1 and inflammatory differentiation of macrophages, which subsequently secrete TNF-α 29, 30, 31. Differentiated macrophages in obesity also release superoxide anions, which further contribute to local vascular dysfunction by diminishing availability of NO (32).

The functional damage we observe in PVAT and its recovery after bariatric surgery are entirely consistent with this hypoxia/inflammation in fat hypothesis. Thus, in our previous studies we observed adipocyte hypertrophy and increased TNF staining in adipose tissue associated with loss of PVAT vasodilatory function. We now show that, after bariatric surgery, there is restoration of PVAT vasodilatory capacity to a degree that is similar to that observed in healthy non-obese participants. It is interesting to note that this functional restoration of the PVAT occurs even though the patients are still severely obese. The improvement in PVAT function after surgery is associated with smaller adipocytes (in the context of a dramatic weight loss), a reduction in PVAT TNF, and increased local adipose tissue NO bioavailability. Our findings are consistent with other studies that have shown significant reductions in macrophage numbers as well as monocyte chemoattractant protein-1 and hypoxia inducible factor-1α in the stromal vascular fraction of white adipose tissue after bariatric surgery (33). Also, importantly, we show that the functional vasodilatory improvement in the subcutaneous fat is due to restitution of PVAT-derived adiponectin and NO bioavailability.

The functional improvement of PVAT after bariatric surgery was independent of the vascular endothelium, because all arteries in this study were denuded of endothelium. Also notably, there were no differences in constriction of arteries devoid of fat to norepinephrine before and after surgery. Taken together, these observations indicate that the improvement in adipose-vascular coupling after surgery was due to improvements in the vasodilating capacity of the PVAT rather than changes to isolated arterial contractility or endothelial function.

### Study limitations

There are a number of limitations to this study that need to be considered in parallel with the findings. We did not monitor the level of exercise performed by the participants at baseline or after surgery, with respect to our patient selection. At 6 months after surgery, the patients had lost a significant amount of weight and were presumably more mobile. As such, we acknowledge that the improvement in PVAT function might be as a consequence of increased levels of mobility. Second, it would have been preferential to recruit participants who were losing weight as part of a calorie-controlled diet to compare changes to PVAT function due to this intervention. This was not possible within the limitations of our faculty, but we hope to perform this study in the future. Similarly, a drawback of this study is that we were not able to perform a 6-month follow-up biopsy on severely obese patients who received no intervention. However, we assume that there would have been no change in PVAT function over this time. We have studied subcutaneous small arteries under the assumption that all small arteries and the surrounding PVAT share similar properties. A separate consideration is that we have detected changes only to subcutaneous gluteal PVAT after surgery. We did not study the function of PVAT from other anatomical sites (i.e., mesenteric or skeletal). As such, we acknowledge that changes to subcutaneous vascular function might not be reflected in other vascular territories, but previous studies have shown similar remodeling profiles of human cerebral and mesenteric arteries to those seen in arteries taken from gluteal subcutaneous biopsies. Finally, it should be noted that norepinephrine was the only contractile agent used. We acknowledge that it would have been preferable to study a variety of vasoconstrictors. However, it should be noted that studies from other groups have shown that PVAT antagonizes contraction to U46619 (34), angiotensin II 35, 36, phenylephrine 35, 37, and serotonin 35, 36.

## Conclusions

Despite these limitations, however, we believe that we present convincing evidence that PVAT-induced vasodilation of small arteries can be fully restored after bariatric surgery. Furthermore, this restoration of function is due to a reduction in inflammation within the adipose tissue, which leads to increased adiponectin secretion. The data offer an insight into 1 mechanism by which bariatric surgery improves vascular function. We anticipate that further work will harness this observation to improve therapeutic options for the severely obese patient.
