# Supplementary material for: Effects of Bariatric Surgery on Human Small Artery Function: Evidence for Reduction in Perivascular Adipocyte Inflammation, and the Restoration of Normal Anticontractile Activity Despite Persistent Obesity
Source: J Am Coll Cardiol. 2013 Jul 9;62(2):128–35. doi: 10.1016/j.jacc.2013.04.027 (PMC3791397; doi:10.1016/j.jacc.2013.04.027)

**Supplementary Figure 1:** Comparison of vessel constriction pre- and post-surgery

A: There is no difference in constriction of skeletonized to NE before and after surgery (P = 0.07, n=15)

B: There is a significant difference in constriction of vessels with intact PVAT before and after surgery (P < 0.0001, n=15)


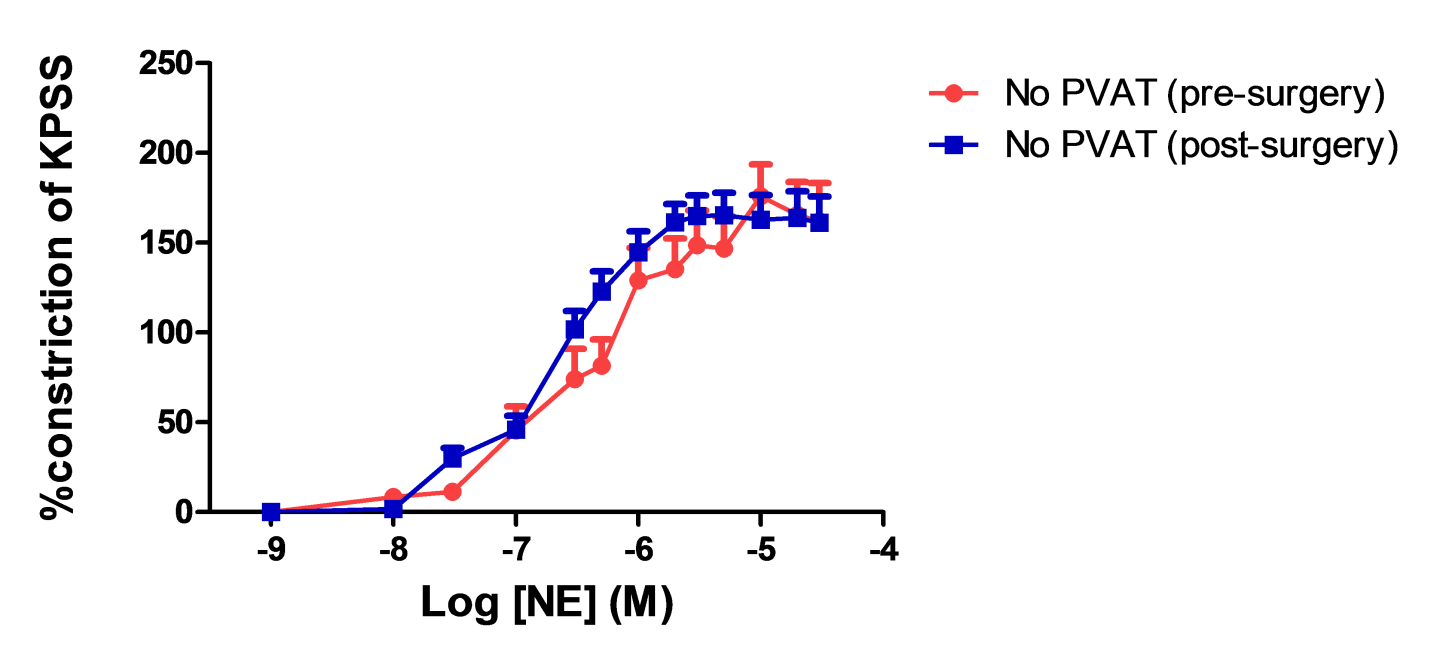


**Supplementary Figure 2:** The change in average adipocyte area post-surgery as compared with baseline correlates with the change in BMI post-surgery (n = 14**,** R^2^ = 0.356, *P* = 0.024)


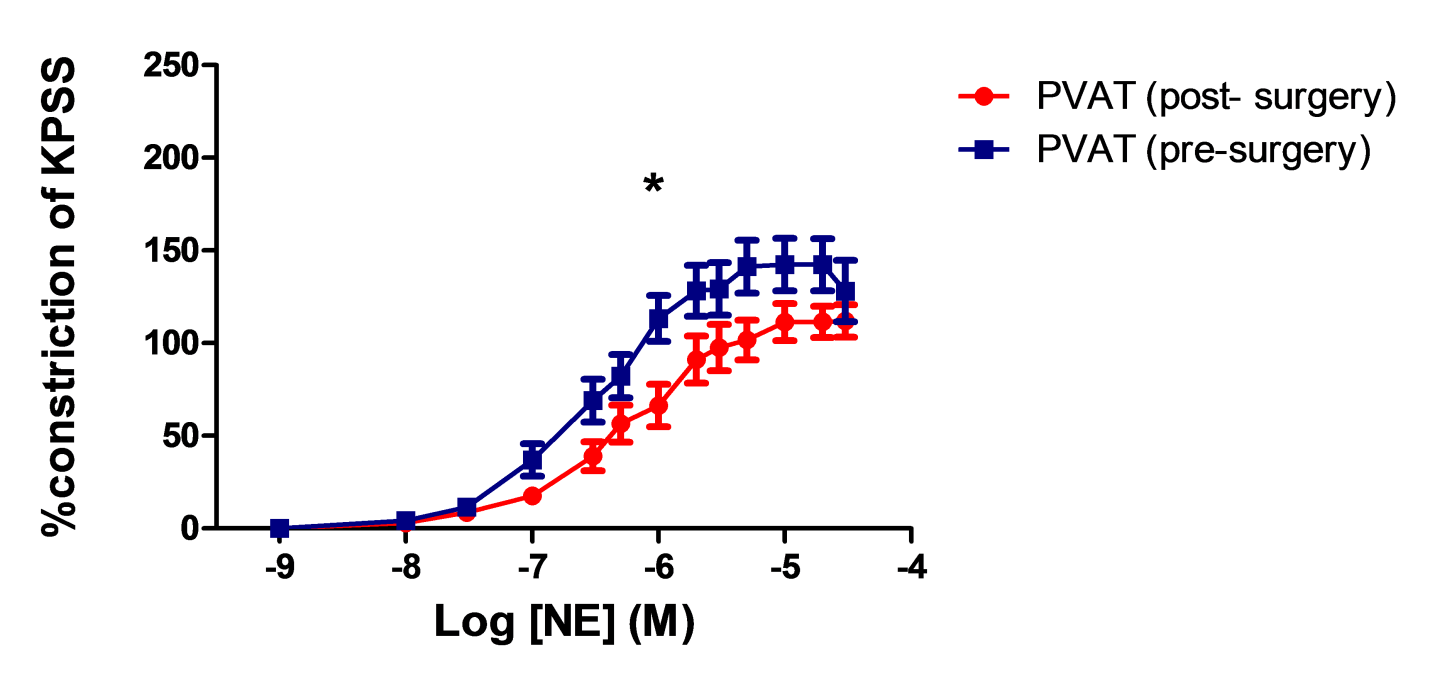

Supplement: Online Figures 1 and 2 [file mmc1.docx]
